# Foot orthoses for the prevention of lower limb overuse injuries in naval recruits: study protocol for a randomised controlled trial

**DOI:** 10.1186/s13047-015-0109-2

**Published:** 2015-09-11

**Authors:** Daniel R. Bonanno, George S. Murley, Shannon E. Munteanu, Karl B. Landorf, Hylton B. Menz

**Affiliations:** 1 Discipline of Podiatry, College of Science, Health and Engineering, La Trobe University, Victoria, 3086 Australia; 2 Lower Extremity and Gait Studies Program, College of Science, Health and Engineering, La Trobe University, Victoria, 3086 Australia

**Keywords:** Randomized controlled trial, Prevention, Orthotic devices, Leg injuries, Military personnel

## Abstract

**Background:**

Foot orthoses are frequently used for the prevention of lower limb overuse injuries but evidence for their effectiveness is limited. The primary aim of this study is to determine if prefabricated foot orthoses reduce the incidence of lower limb overuse injuries in naval recruits undertaking 11 weeks of basic training.

**Methods:**

This study is a participant and assessor blinded, parallel-group, randomised controlled trial. The trial will recruit participants undertaking 11 weeks of basic training at the Royal Australian Navy Recruit School, Cerberus, Victoria, Australia. Participants will be randomised to a control group (flat insole) or an intervention group (prefabricated foot orthosis). Over the 11 weeks of basic training, participants will document the presence and location of pain in weekly self-report diaries. The end-point for each participant will be the completion of 11 weeks of basic training. The primary outcome measure will be the combined incidence of four lower limb injuries (medial tibial stress syndrome, patellofemoral pain, Achilles tendinopathy, and plantar fasciitis/plantar heel pain) which are common among defence members. Secondary outcome measures include: (i) overall incidence of lower limb pain, (ii) severity of lower limb pain, (iii) time to injury, (iv) time to drop-out due to injury, (v) adverse events, (vi) number of lost training days, (vii) shoe comfort, and (viii) general health status. Data will be analysed using the intention-to-treat principle.

**Discussion:**

This randomised controlled trial will evaluate the effectiveness of prefabricated foot orthoses for the prevention of common lower limb overuse injuries in naval recruits.

**Trial registration:**

Australian New Zealand Clinical Trials Registry: ACTRN12615000024549.

## Background

Lower limb overuse injuries are common in people who participate in regular physical activity [1–4]. For example, the incidence of lower limb overuse injuries among long-distance runners and physically active defence members has been reported to range from 19 to 79 % [3, 4]. The most common injuries include medial tibial stress syndrome, patellofemoral pain, Achilles tendinopathy and plantar fasciitis/plantar heel pain [1–3]. Lower limb injuries result in lost training time, incur financial costs, adversely affect an individual’s physical and mental health, and increase the likelihood of stopping physical activity [5].

Given the high incidence and detrimental effects of lower limb overuse injuries, interventions that are effective at preventing injuries are likely to have a significant positive impact on health-related quality of life [5]. However, a recent systematic review concluded that there is only weak evidence to support the use of interventions, including foot orthoses, to prevent lower limb injuries and more high quality trials are needed [6].

Foot orthoses are commonly used [7], having been shown to be beneficial for treating several musculoskeletal disorders of the lower extremity [8–10], as well as reducing the incidence of shin splints [11] and femoral stress fractures [12]. Although the specific mechanism of action by which foot orthoses provide benefits remains unclear, there is evidence that they alter kinematics, kinetics, and/or muscle activity of the lower limb [13]. Evidence to support the use of foot orthoses to prevent lower limb injuries is limited, though, as existing trials are generally of poor methodological quality [9, 14].

Therefore, this study aims to determine if prefabricated foot orthoses reduce the incidence of common lower limb overuse injuries in naval recruits undertaking 11 weeks of basic training.

## Methods

### Design

The Australian Navy Cerberus Orthotic Research (ANCOR) study is a participant and assessor, parallel-group blinded, randomised controlled trial. Participants will be randomised to a control group (flat insole) or an intervention group (prefabricated foot orthoses). To ensure allocation concealment, permuted block randomisation with random block sizes, stratified by sex, will be undertaken using an interactive voice response telephone service provided by the NHMRC Clinical Trials Centre (University of Sydney, New South Wales, Australia). The end-point for each participant will be the completion of their 11 week basic training programme. The location of the trial will be at the Royal Australian Navy Recruit School, Her Majesty's Australian Ship (HMAS), Cerberus, Victoria, Australia (HMAS Cerberus). The trial has been registered on the Australian New Zealand Clinical Trials Registry (ACTRN12615000024549). The findings from the trial will be reported according to the Consolidated Standards of Reporting Trials (CONSORT) statement [15, 16].

### Ethical approval

Ethical clearance for this project was provided by the Australian Defence Human Research Ethics Committee (protocol number: 764–14) and the La Trobe University Faculty Human Ethics Committee (protocol number: FHEC 14/250). All participants will provide written informed consent prior to recruitment. Ethical standards will adhere to the National Health and Medical Research Council National Statement [17] and the World Medical Association’s Declaration of Helsinki [18].

### Participants

Participants will be naval recruits from the Australian Defence Force (ADF). The minimum age of entry for naval recruits is 17 years, with the maximum age being generally three to six years before the compulsory retirement age of ADF members (60 years) [19]. Prior to undertaking basic training, naval recruits must pass a pre-entry medical and fitness test [19]. Fitness requirements include a minimum of 20 sit-ups and a multistage 20 m shuttle run test [20] score of 6.1. The multistage 20 m shuttle run test involves running between two points that are 20 m apart and doing so within a series of pre-recorded audible beeps, with time between beeps decreasing as the test proceeds [20]. Achieving a shuttle run score of 6.1 equates to 43 shuttles or a total of 860 m in 5 min and 15 s. In addition, female and male recruits are required to perform a minimum of 6 and 15 push-ups, respectively. The maximum allowable body mass index (BMI) is 32.9 kg/m^2^; and recruits with a BMI less than 18.5 kg/m^2^ may be deemed temporary unfit by medical staff, with this decision determined in consideration of the recruit’s general and medical fitness.

**Fig. 1:**
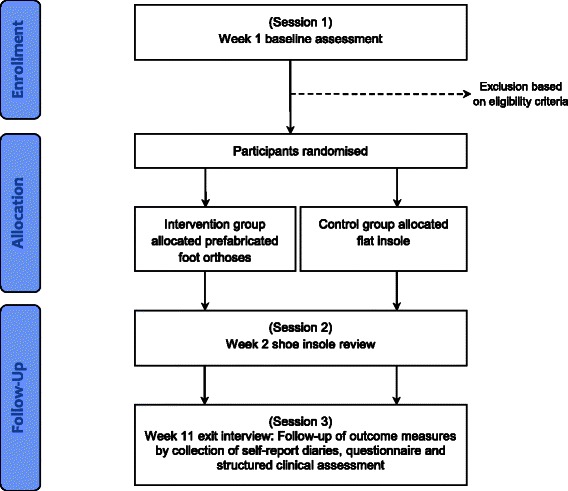
Flow of participants through the trial

All naval recruits undertaking 11 weeks of basic training at HMAS Cerberus will be invited to participate in the trial. Consecutive cohorts of recruits will be invited to participate until the pre-specified sample size (see below) is achieved. Participants will be excluded if they use foot orthoses or have a lower limb injury (causing pain of least 30 mm on a 100 mm visual analogue scale when at worst [21, 22]) at the time of recruitment. All recruits will be provided information about the study at the commencement of their 11 week training programme. Recruits interested in participating in the study will be invited to attend an initial appointment within their first week of training (session 1). Figure 1 outlines the flow of participants through the trial.

### Sample size

A prospective sample size calculation has estimated that 306 participants (i.e. approximately 153 participants per group) are required to provide 80 % power to detect a clinically worthwhile difference of a 50 % reduction in injury in the intervention group (alpha set at 5 %). The sample size for the study was calculated assuming: (i) a combined incidence of injury of 30 % for medial tibial stress syndrome, patellofemoral pain, Achilles tendinopathy, and plantar fasciitis/plantar heel pain, and (ii) a drop-out of 20 %. The estimated incidence of lower limb injury was based on published data from studies investigating injury among Defence members completing basic training [2, 23, 24]. The drop-out rate for this trial was conservatively selected at the higher end of previous trials investigating the use of foot orthoses for the prevention of injuries in defence settings [25, 26], while also taking into account the completion rate of naval recruits undertaking the 11 weeks of basic training at HMAS Cerberus in the previous 12 months.

### Interventions

Interventions will be provided by experienced podiatrists who are professionally registered with the Podiatry Board of Australia. Prior to the study commencing, the assessors will attend a minimum of two training sessions. A detailed manual outlining study procedures will be provided to all assessors. The training sessions will be conducted by the chief investigator (DRB) who has 10 years of clinical and research experience using foot orthoses for musculoskeletal conditions.

**Fig. 2:**
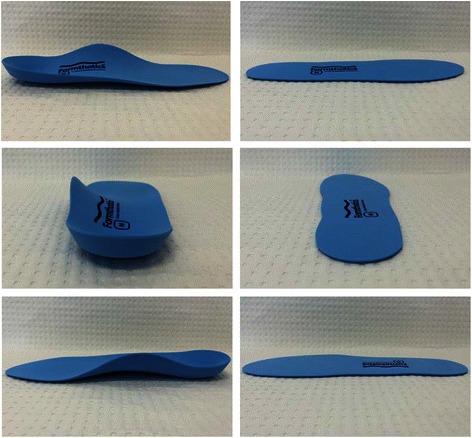
The prefabricated foot orthosis (left) and flat insole (right) prior to being heat moulded to a participant’s foot. Top panels show lateral view, middle panels show posterior view and lower panels show medial view

**Fig. 3:**
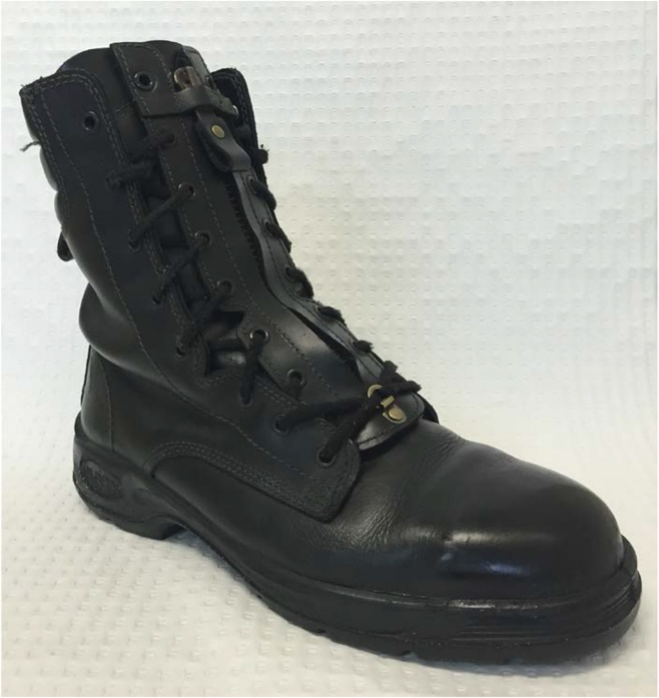
Oliver Footwear Pty Ltd Structural Fire Fighter Boot (Model Number:20292)

Participants will be randomised to one of two groups: (i) a *control group* that will receive a pair of 3 mm flat insoles, or (ii) an *intervention group* that will receive a pair of Formthotics® prefabricated foot orthoses (Model: Original Single Medium) (Fig. 2). Both interventions, collectively referred to as shoe insoles, will: be manufactured by the same company (Foot Science International, Christchurch, New Zealand), be full-length insoles made from the same material (140 kg/m^3^ single density, closed cell polyethylene foam), and will have the same branding (i.e. company logo). The only difference between the two interventions will be the geometry and level of support that the shoe insoles provide (Fig. 2). To maintain blinding of participants, the participants will only be advised that they will receive one of two types of ‘shoe insole’ during the study. The allocated shoe insoles (flat insole or prefabricated foot orthosis) will be placed in the participant’s footwear and heat moulded to the participant’s feet and footwear. To achieve this, the participants will receive their footwear containing their heated insoles and they will be required to stand in them to enable the insoles to mould to their feet, as per the manufacturer’s instructions (Foot Science International, Christchurch, New Zealand). For the 3 mm flat insole, this moulding process will be one largely of slight compression of the material under weightbearing areas of the foot (i.e. it will not provide support to the arch of the foot or substantial contouring around the heel). Each participant will have their shoe insoles fitted to their athletic shoes and Defence-issued boots (i.e. two pairs of shoe insoles per participant) to maximise convenience and adherence to the interventions. All recruits will receive the same Defence-issued boots (Oliver Footwear Pty Ltd Structural Fire Fighter Boot, Model Number 20292) at the beginning of their 11 weeks of basic training (Fig. 3).

### Data collection sessions

Participants will attend three data collection sessions: baseline (session 1), week 2 (session 2) and week 11 (session 3).

#### Session 1 (baseline)

An initial assessment will be performed to determine the eligibility of participants. Injury history, general demographic data, physical assessments and anthropometric measures will be collected. This will include participants’ age, sex, waist and hip circumference, height and weight, foot posture [27], ankle joint dorsiflexion [28, 29], and a rating of lumbopelvic stability [30]. General health status will be determined using the Short-Form-12 Version 2 questionnaire [31] and physical activity (domestic, travel, work and recreation) in the previous four weeks will be measured using the Recent Physical Activity Questionnaire (RPAQ) [32]. Participants will also undergo a brief physical examination to determine if the clinical features used for the diagnosis of the four most expected lower limb injuries (Table 1) are present. Fitness information (e.g. multistage 20 m shuttle run test results) collected by Defence during the initial weeks of basic training will also be obtained for each participant.

**Table 1:** Clinical features used for the diagnosis of the four most expected lower limb injuries

Injury	Clinical features
Medial tibial stress syndrome	Diffuse pain or oedema along the posteromedial border of the tibia;
	Pain spread over a minimum of 5 cm;
	Pain occurs with activity and lasts for at least a few hours post activity;
	Diffuse discomfort produced with palpation along the posteromedial border of the tibia, with discomfort confined to this region; and
	No history of paraesthesia.
Patellofemoral pain	Insidious onset of peripatellar or retropatellar knee pain;
	Pain on patellofemoral joint compression or resisted isometric quadriceps contraction at 30 degrees of knee flexion; and
	Peripatellar or retropatellar knee pain being provoked by at least two of the following activities: running, hopping, walking, marching, squatting, stair negotiation, prolonged sitting, or kneeling.
Achilles tendinopathy (midportion)	Insidious onset of pain located within 2 to 7 cm proximal to the insertion on the calcaneus; and
	Pain is reproducible with palpation of the Achilles tendon within 2 to 7 cm proximal to the insertion on the calcaneus; and
	Pain most noticeable after an extended period of rest and aggravated with activity.
Plantar fasciitis/plantar heel pain	Presence of pain in the plantar heel or medial arch;
	Pain is worse after rest but eases with mild activity;
	Pain is generally worse with prolonged standing or activity; and
	Pain is reproducible with palpation of the medial tuberosity of the calcaneus and/or along the plantar fascia.

Once all baseline measures have been taken, a therapist (separate investigator) located in an adjacent room (with no view of the participants) will allocate participants to one of the two groups (using the allocation system outlined in the manuscript). The therapist will fit and heat the allocated insoles into the participant’s footwear. Once the insoles have been heated, the therapist will place the participant’s footwear on a table outside of their room for the blinded assessor. The assessors, unaware of the insole allocation, will collect the participant’s footwear and advise the participants to wear their footwear for several minutes to allow moulding. Following this, participants will be asked to rate the comfort of their shoes after a brief period of wear (see the ‘outcome measures’ section for more detail). In addition, all participants will be issued with: (i) an information sheet informing them of the recommended wearing-in protocol and how to care for their shoe insoles, and (ii) an individual diary for participants to self-report pain levels over the 11 weeks of basic training. The self-report diaries will consist of body [33] and foot [34] pain drawings, which have been shown to have good to excellent inter- and intra-rater reliability of scoring the location of pain [33, 35]. Each week, participants will be required to indicate the presence and location of pain by shading within the outlines of the pain drawings. If pain is reported, participants will also be required to indicate the usual and worst pain experienced during the previous week on two separate 100 mm visual analogue scales (VAS) (see the ‘outcome measures’ section for more detail). At the completion of the initial assessment, participants will be invited to attend a review consultation the following week (session 2).

#### Session 2 (week 2)

At this session an assessor will be available to review all participants who are not finding their insoles comfortable. If participants report that the allocated insoles are uncomfortable, the insoles will initially be inspected to ensure that they fit the shoes and foot properly and will be re-moulded as per session 1. If adequate comfort is still not achieved, the section of the insole causing the discomfort will be identified and modified (either by spot heating or by grinding material away in the area of concern). The assessor will attempt to remove the least amount of material required to achieve comfort.

#### Session 3 (week 11)

An exit interview will be conducted with an assessor that is blinded to group allocation in the final week of training (week 11). This session will be used to collect self-report diaries and confirm their accuracy and completeness with participants. In addition, if lower limb pain is being experienced by the participants at this session, the assessor will attempt to determine the presence of medial tibial stress syndrome [23], patellofemoral pain [36], Achilles tendinopathy [37], and plantar fasciitis/plantar heel pain [21, 38]. Finally, injury data will be cross-checked against ADF medical records.

All participants will remain blinded to their group allocation and will have a similar experience at all sessions regarding time spent with assessors, advice provided, and data collected. That is, the only difference between the groups will be the type of shoe insole received.

### Definition of injuries

For the purpose of this trial, an injury will be defined by the presence of pain that scores at least 30 mm on a 100 mm visual analogue scale when at its worst [21, 22]. Due to the lack of evidence regarding the diagnostic accuracy of the lower limb injuries of interest, the diagnosis of medial tibial stress syndrome [23], patellofemoral pain [36], Achilles tendinopathy [37], and plantar fasciitis/plantar heel pain [21] will be determined using a pragmatic approach via clinical assessments based on definitions described in previous studies (Table 1).

Over the 11 weeks of basic training, participants will document the presence and location of pain on pain drawings in their weekly self-report diaries. The pain drawings will identify the presence and location of pain in the body or feet, but they will not provide a specific diagnosis regarding the cause of pain. Once the self-report diaries have been submitted at the exit interview, the assessors will use a transparent overlay divided into multiple body areas to determine pain location.

### Outcome measures

The *primary outcome measure* will be the combined incidence of the four common lower limb injuries (medial tibial stress syndrome, patellofemoral pain, Achilles tendinopathy, and plantar fasciitis/plantar heel pain) as determined at medical appointments and by assessors at the exit interview (week 11).

*Secondary outcomes* include:

(i) the overall incidence of lower limb pain as determined by pain drawings in self-report diaries, measured at week 11;
(ii) the severity (usual and worst) of over-use lower limb pain as determined by the 100 mm VAS in self-report diaries, measured at week 11;
(iii) the time (in days) to lower limb injury and pain as determined by self-report diaries and navy medical records, measured at weeks 1 to 11;
(iv) time to drop-out from injury (in days) as determined by navy administration records, measured at weeks 1 to 11;
(v) the type, frequency and severity (mild, moderate or severe) of self-reported adverse events (such as new pains, shoe/insole discomfort and blisters) will be obtained from the self-report diaries, measured at weeks 1 to 11;
(vi) lost training days as determined by navy administration and medical records, measured at weeks 1 to 11;
(vii) shoe comfort will be measured using a 100 mm visual analogue scale, measured at baseline and week 11;
(viii) general health status will be determined using the Short-Form-12 (SF-12) Version 2 questionnaire [31], measured at baseline and week 11.

Shoe comfort will be determined using a 100 mm visual analogue scale, which has been shown to have good reliability [39]. The left end of the scale (0 mm) is labelled “not comfortable at all” and the right end of the scale (100 mm) is labelled “most comfortable imaginable”. Participants will be instructed to place a mark on the scale that represents their perceived comfort rating. Similarly, pain will be determined using a 100 mm visual analogue scale. To indicate the range of pain to participants, the left end of the scale (0 mm) is labelled “no pain” and the right end of the scale (100 mm) is labelled “worst pain imaginable”. The VAS has been shown to be a valid [40] and reliable [39] measure of pain.

### Evaluation of adherence

Adherence to the allocated shoe insoles will be documented by participants each week in self-report diaries. Participants will record the number of hours per day and days per week the shoe insoles have been worn [41]. In addition, adherence to the shoe insoles will be confirmed by a blinded assessor at session 3.

### Adverse events

Adverse events will be documented by participants in weekly self-report diaries. An adverse event will be considered any harmful or unpleasant outcome for which there is a known or plausible association with the shoe insoles and those for which there is none [42].

### Statistical analysis

Statistical analysis will be performed using SPSS version 22.0 or later (SPSS Corp, Chicago, Ill, USA) using the intention-to-treat principle, where all randomised participants will be included in the final analyses. The endpoint will be the completion of the 11 weeks of basic training for each participant. Multiple imputation will be used to replace any missing data using five iterations, with age, and group allocation as predictors [43]. The exception will be for the variable adverse events where no data substitution will be applied.

Participant characteristics and baseline data will be summarised by descriptive statistics. Distribution of continuous variables will be checked for normality and transformation will be carried out if necessary prior to the use of parametric statistics. Outcome data will be analysed according to a pre-planned protocol. Differences in the primary and secondary outcome measures between the two groups will be compared.

The differences between groups for the primary outcome measure of the combined incidence of the four common lower limb injuries (medial tibial stress syndrome, patellofemoral pain, Achilles tendinopathy, and plantar fasciitis/plantar heel pain) and the secondary outcome measure of the incidence of lower limb pain (overall and body region specific) during the 11 week training period will be compared using incidence rate ratios.

In regard to the other secondary outcomes, differences between groups for continuous outcome measures (severity of pain, lost training days and shoe comfort) will be analysed using independent t-tests. The time to lower limb injury and pain and drop-out will be compared using Cox proportional hazards ratios. Adherence of the shoe insole interventions will also be analysed using independent *t*-tests. The difference between groups for the outcome measure of health status (SF-12) will be compared using analysis of covariance (ANCOVA) with baseline scores and intervention group entered as independent variables [44].

95 % confidence intervals will also be provided to indicate precision of point estimates. We will also conduct hypothesis tests where appropriate, which will be considered significant if *p* < 0.05. Effect sizes will be determined using standardised mean differences (Cohen’s *d*) for continuous data.

## Discussion

This randomised controlled trial is being conducted to determine the effectiveness of prefabricated foot orthoses for the prevention of lower limb overuse injuries. Numerous clinical trials have previously investigated whether foot orthoses prevent injuries; however they are generally of poor methodological quality [9, 14]. Of particular note, the majority of previous trials lacked participant and assessor blinding, and few compared the intervention to a control intervention [9, 14], which is likely to bias or confound the findings [45, 46].

The foot orthosis selected for this trial were chosen for four main reasons. Firstly, prefabricated foot orthoses were considered more practical than customised foot orthoses (made from a cast or mould of the foot) as they can be issued to participants immediately, which is preferable in defence force populations. Secondly, prefabricated foot orthoses are relatively inexpensive compared to customised orthoses. This is an important consideration, as if this study demonstrates that foot orthoses can prevent injuries, cost effectiveness is likely to be a factor when deciding whether foot orthoses become standard issue for defence force recruits. Thirdly, the specific prefabricated foot orthosis (Formthotics®, Foot Science International, Christchurch, New Zealand) was selected as similar devices have been used in large randomised trials with few orthotic-related adverse events being reported [11, 47, 48]. The selected orthosis is made from polyethylene foam and a similar trial reported fewer orthotic-related participant drop-outs in those assigned to an orthosis made from a foam-based material (9 %) compared to a semi-rigid plastic (37 %) [26]. Such factors are important because minimising adverse events and ensuring the orthosis is comfortable is particularly important in this trial as the naval recruits will undertake rigorous activity within days of receiving their allocated shoe insoles. Fourthly, the selected orthosis is commercially available and widely used in clinical practice, which should enable them to be readily utilised as a preventative intervention if they are indeed found to be effective in this trial.

A distinguishing feature of this trial is the use of a flat insole as a control intervention. The inclusion of a flat insole as a control intervention assists with accounting for non-intervention effects not directly related to an experimental intervention (e.g. placebo effect, Hawthorne effect, natural resolution, etc.) [45, 49]. Similar flat insoles have been perceived by participants as being equally credible and expected to provide similar benefits as foot orthoses [50], which is likely to aid blinding and minimise confounding factors such as resentful demoralisation among participants not receiving the prefabricated orthosis [46]. The potential effects of resentful demoralisation cannot be totally mitigated by the use of a control intervention as the participants will reside within the same barracks and there is the possibility that they will compare insoles and notice differences between them. However, as all insoles have the same branding, are made from the same material and will be heat moulded to the participant’s feet they are intended to look as similar as possible. It is worth noting that the flat insole used in this study is best considered as a ‘sham’ and not a true placebo, as it is likely to provide some mechanical (e.g. plantar pressure) effects to the foot, as shown in previous studies that used similar materials [50, 51]. In consideration of these potential effects, a flat insole was selected as the control intervention as similar insoles, whether flat or contoured, have been shown to provide the same mechanical effects as a shoe alone in the midfoot region [50]. This was considered important as one of the major modes of how foot orthoses are proposed to provide benefits is through increased loading of the plantar-medial midfoot [52]. Clearly, the decision of what control insole to use is a balance between minimising non-interventions effects and avoiding participants experiencing resentful demoralisation.

The eligibility criteria do not require participants to have a particular foot type. Although foot orthoses are most frequently used to treat overuse conditions associated with excessive foot pronation [8, 47, 53], orthoses have been shown to benefit a range of different foot postures including pronated and supinated feet [51, 53]. In addition, foot orthoses effect lower limb kinematics, kinetics, and muscle activity with the specific mechanism of action remaining unknown [13, 54]. As such, any benefits foot orthoses provide may result from one or all of these mechanisms and not necessarily be related to foot posture [24]. Most importantly, it is not currently possible to identify individuals most likely to benefit from the prophylactic use of foot orthoses using particular characteristics such as foot posture.

Finally, this trial has been designed to optimise its scientific rigour, which will overcome some of the limitations of previous trials. Some strengths of the trial include the use of allocation concealment, participant and assessor blinding, blinded data entry, adhering to the intention-to-treat principle to analyse data and the use of a control intervention. In addition, as the trial is being conducted in a defence force setting, variables such as access to medical care, training loads, diet, sleep, clothing and footwear will be largely standardised. However, there is one potential limitation with this trial, and that is the generalisability of the population to be studied. Participants will be relatively homogenous as they will predominantly be healthy young adults that are enrolled in a relatively intense physical training programme. Accordingly, the findings of this trial will need to be generalised to the broader community in consideration of such strengths and limitations.

## Conclusions

This randomised controlled trial will evaluate the effectiveness of foot orthoses for the prevention of lower limb injuries. Specifically, it will determine whether prefabricated foot orthoses are more effective than flat insoles at reducing the incidence of common overuse lower limb injuries in naval recruits undertaking 11 weeks of basic training.

## Trial status

Recruitment of participants commenced on 28th January 2015 and final results are expected to be available in 2016.
